# Are Mesenchymal Stem Cells So Bloody Great After All?

**DOI:** 10.5966/sctm.2016-0026

**Published:** 2016-09-09

**Authors:** Stephanie J. Marfy‐Smith, Claire E. Clarkin

**Affiliations:** ^1^ Biological Sciences, University of Southampton, Southampton, United Kingdom

## Abstract

This Perspective discusses some activities of mesenchymal stem cells (MSCs) in the context of angiogenesis, focusing on contrasting effects that could call into question the extent to which MSCs can be used clinically in the future. We report on the antiangiogenic/antiproliferative effects of specific MSC populations (including bone marrow MSCs), their paracrine activity, tissue heterogeneity, and endothelial cell interactions. Also discussed are what could lead to contrasting effects of the influence of MSCs in regulating angiogenesis, pointing to some negative effects of these cells. In conclusion, this article highlights important aspects of MSC behavior within the perspective of translational medicine applications. Stem Cells Translational Medicine *2017;6:3–6*

**Significance Statement**

Multipotent mesenchymal stem cells (MSCs) can be extracted from virtually every organ and tissue in the body. Although they have previously been shown to be an important source of blood vessel‐attracting factors, useful for tissue repair and regenerative medicine, recent studies have found that specific MSC populations can also produce factors that inhibit blood vessel growth. Abnormal vascularization is associated with the progression of many diseases, and identification of these unique blood vessel‐inhibiting MSCs has highlighted a potential source of cytotoxic factors that could be used to control pathological angiogenesis, for example, tumors.

## Introduction

Past research efforts describing the beneficial effects of mesenchymal stem cells (MSCs) clinically originally focused upon their multipotency and capacity for self‐renewal. However, today we now know that MSCs also produce a broad spectrum of proangiogenic factors that can directly facilitate the proliferation and migration of endothelial cells (ECs) and contribute to the recruitment of endothelial progenitor cells 1. As a consequence, MSCs may exert indirect benefits via the production of growth factors to positively influence blood vessel infiltration and improve rates of tissue regeneration and repair.

Advantageous effects of MSCs on vascular function have been described widely in animal and clinical studies, including ischemia, in which bone marrow (BM)‐derived MSCs enhance limb perfusion, increase blood vessel density, and improve overall limb function in a murine model 2. Further evidence has demonstrated the therapeutic benefits of BM 3, adipose 4, and pulp 5 MSCs in a rat model of myocardial infarction in which MSC transplantation significantly improved ventricular function and was associated with increased angiogenesis. In stroke, the initial idea of MSC transplantation was by the potential transdifferentiation of MSCs into glial cells and neurons, but the beneficial effects actually appear to be a result of MSC‐mediated enhancement of endogenous angiogenesis 6. Cotransplantation of murine kidney MSCs with islets of Langerhans has been shown to significantly improve clinical outcomes of diabetic mice by increasing islet graft revascularization following transplantation 7. Combination therapies of MSCs and polymeric growth factor release scaffolds tailored to promote angiogenesis and osteogenesis are also under evaluation and development to actively stimulate bone regeneration (reviewed by Kanczler and Oreffo 8). MSCs are generally thought to influence EC function by the paracrine production of proangiogenic factors including vascular endothelial growth factor (VEGF), hepatocyte growth factor, basic fibroblast growth factor, insulin‐like growth factor‐1, transforming growth factor‐β (TGF‐β), platelet‐derived growth factor (PDGF), angiopoietin, interleukin‐6 (IL‐6), and monocyte chemotactic protein‐1. Exosomes released by MSCs are also thought to influence angiogenesis by transferring genetic materials and angiogenic molecules. ECs also have a reciprocal capacity to modulate the behavior of mesenchymal cells, for example, by the expression of growth promotors and inhibitors such a Notch 9. And there is accumulating evidence to suggest the BM‐MSCs may also have promoting effects on hematopoietic stem cells (HSCs) with engraftment and repopulation in several studies demonstrating that cotransplantation of human MSCs and HSCs results in increased hematopoietic recovery in animal models and humans 10 11 12.

In contrast to the body of accumulated work detailing positive effects of MSCs on angiogenesis, several recent studies have emerged that describe an apparent detrimental influence of MSCs on EC function. Such contrasting effects could question the extent to which MSCs could be used clinically, and the reported anti‐angiogenic effects of specific MSC populations will be the focus of this Perspective.

## Antiangiogenic Actions of Bone Marrow‐Derived MSCs

Because of their accessibility, bone marrow (BM)‐derived MSCs are probably the best characterized MSC population and the most commonly used MSC in clinical practice. One of the most heavily researched areas with regard to effects of MSCs on vascular EC function clinically is with reference to tumorigenesis. Contrasting effects of the influence of MSCs in regulating angiogenesis have been described in the context of tumor growth, which is unsurprising given the complexity of the disease, the broad cellular heterogeneity of the tumor itself, and its reliance upon the vasculature. BM‐MSCs have been shown to recruit ECs to induce angiogenesis in stable tissue 13 as well as in tumors 14, raising the possibility that MSCs may promote tumor growth. However, by contrast, intravenously injected MSCs were found to abrogate growth of the Kaposi sarcoma 15, suggesting that MSCs could also possess cytotoxic properties.

A concentration‐dependent inhibition of angiogenesis has been reported in melanoma, with rat BM‐MSCs. Using in vitro capillary cultures, it was shown that addition of MSCs caused a dose‐dependent effect on EC cytotoxicity that was attributable to MSC:EC gap junction communication and the production of MSC‐derived reactive oxygen species. The combined effect of these responses resulted in capillary destruction. Furthermore, in an in vivo melanoma model, the rat BM‐MSCs inhibited tumor growth by abrogating growth of the tumor vasculature, and these results demonstrate a novel property of MSCs, namely, as cytotoxic agents that can inhibit the formation of capillary networks 16. Following on from this study, physiologically, the large numbers of MSCs that would be required for such cytotoxic effects are unlikely to be achieved in capillary beds with normal blood flow, because the cells are likely to be flushed away. However, cytotoxic effects might become evident to the extent that administered MSCs aggregate in, for example, the reticulo‐endothelial system, such as in the liver and spleen.

A further example of the antiproliferative activity of human MSCs on tumor cells of hematopoietic origin was reported by Ramasamy et al., who showed that BM‐MSC produced the transient arrest of tumor cells in the G1 phase of the cell cycle in vitro 17. A coinjection of tumor cells and BM‐MSCs, however, resulted in an increased incidence of tumor growth in immunodeficient mice. The authors concluded that the discrepancy between the in vitro and in vivo findings would be due to development of a cancer stem cell niche after cotransplantation of MSC in which the tumorigenicity can be augmented 17. Coadministration of human BM‐MSCs and glioma cells also significantly reduced tumor size and vascular density, with MSCs downregulating the expression of angiogenic molecules 18 in glioma cells and BM‐MSC glioma cocultures showing reduced expression of PDGF‐B and IL‐1β. It was proposed that the MSCs may exert their antitumor effect through the downregulation of the PDGF/PDGFR axis, which is critical in the regulation of glioma angiogenesis. Tumor cells play a decisive role in mutual cross‐talks between the diverse heterogeneity of cell types in the tumor microenvironment 19, suggesting the possibility of dual, contradicting effects of MSCs in tumor neo‐angiogenesis dependent on tumor origin. For example, in contrast to the proangiogenic effects of MSCs described in “normal” tissue, it appears evident in specific tumor microenvironments that MSCs alter their production of proangiogenic growth factors and mediators.

Outside of tumorigenesis, an antiangiogenic and anti‐inflammatory action of rat BM‐MSC lines has also been described in corneal wound healing 20. Investigations were undertaken to determine whether transplantation of MSCs could improve ocular surface reconstruction by modulating corneal inflammation and angiogenesis in mice with a chemical burn. Surprisingly, the authors described a rapid regression of new vessels in the MSC or MSC‐conditioned media group, which was consistent with other studies 21, 22. To examine this further, the authors measured the expression of molecules known to be involved in corneal angiogenesis and found an upregulation of antiangiogenic thrombospondin‐1 and downregulation of proangiogenic matrix metalloproteinase.

## Tissue Heterogeneity and Endothelial Cell Interactions

To date, MSCs have now been successfully isolated from a number of other organs including brain, liver, kidney, lung, muscle, thymus, pancreas, skin, lung umbilical cord, and placenta 23. It has been proposed that the capacity to modulate the formation of the vasculature is a ubiquitous property of all MSCs, irrespective of their anatomical location. In one study, MSCs were isolated from four murine tissues, including bone marrow, white adipose tissue, skeletal muscle, and myocardium 24. The authors described that all four MSC populations secreted a plethora of proangiogenic factors that induced proliferation, migration, and tube formation of endothelial colony forming cells (ECFCs). In vivo, coimplantation of these MSCs with ECFCs into mice generated an extensive network of blood vessels, with ECFCs specifically lining the lumens and MSCs occupying perivascular positions. But importantly, the authors concluded that there were no differences among all four different populations of MSCs evaluated.

In contrast, others have described potent differences existing between populations of tissue derived‐MSCs and their capacity to regulate the vasculature. Kern et al. compared the proliferative capacity of MSCs isolated from BM, adipose tissue, and umbilical cord, and analysis of BM‐MSCs and umbilical cord vein MSCs that followed showed that the former had higher expression levels of genes associated with osteogenic differentiation, whereas the latter exhibited a higher expression of genes involved in angiogenesis 25. It was also demonstrated that adipose‐derived MSCs exhibit greater angiogenic potential in comparison with BM‐MSCs 26 and may be more effective in cardiovascular pathologies associated with ischemia.

Consistent with MSCs from different tissue sources exhibiting unique behaviors, an endogenous population of MSCs present in islets of Langerhans has recently been identified that exerts potent detrimental effects on EC survival in vitro 27. Upon isolation and expansion, human islet MSCs expressed higher levels of proangiogenic VEGF when compared with human BM‐MSCs. However, after coculture with human microvascular ECs, islet MSCs exerted a rapid negative effect on EC viability, inducing EC apoptosis both following direct cell:cell contact and via the production of soluble mediators. When TGF‐β signaling via activin‐like kinase‐5 activity was inhibited during coculture, EC survival was maintained, highlighting that MSC‐derived growth factors were responsible for this effect.

## Spatial Influence on MSC‐Angiogenic Potential

Differential impacts of MSCs on EC function have also been described within the same study using two‐dimensional (2D) and three‐dimensional (3D) culture systems. In a 2D culture system of ECs (human umbilical vein endothelial cells) and human BM‐MSCs, a cell count was performed, and after 9 days of coculture the number of viable ECs was significantly reduced in the presence of MSCs. Monocultured ECs showed a typical cobblestone‐like morphology after 1 week's 2D culture in vitro, and with the addition of BM‐MSCs a less proliferative phenotype could be observed in which the ECs did not organize into microvascular networks in the presence of MSCs at this ratio. In order to determine the influence of BM‐MSCs on EC quiescence, the expression of CD31 and vWF was analyzed whereby in EC:MSC constructs, a downregulation of both markers was found after 1 week's culture in vitro, as well as after 1 week's in vivo implantation. In contrast, 3D cell/scaffold constructs induced a higher vascular density than did control scaffolds, whereas the highest density of capillaries was achieved through coseeding of ECs and MSCs 28.

## Conclusion

Although MSCs have previously been given much attention because of their positive effects upon EC growth and angiogenesis in instances such as tissue transplantation or regeneration, it now appears that communication with vasculature by MSCs is complex and tissue specific. Evidence strongly supports a perivascular origin for MSCs, and previous results have demonstrated that MSCs can stabilize and maintain vascular structures in vivo 29. Indeed, a landmark publication has presented a large body of work that defines and validates both the in situ and in vitro links between MSCs and perivascular cells or pericytes 30. An increased understanding of the origin of MSCs and their contribution to blood vessel function as perivascular cells in vivo should help clarify why MSCs appear to exert such differential effects on EC function. Indeed, it is well established that in vivo the vasculature is highly heterogeneous, and the endothelium can mold itself and its behavior to the functional requirements of the underlying tissue; therefore, it is entirely plausible that pericytes and MSCs in vivo could exert similar levels of heterogeneity dependent on the function of the tissue in which they exist.

Current evidence suggests that MSCs can exert variable immunomodulatory effects that are also dependent on the local microenvironment or disease status; for example, MSCs decrease the Th1 response in patients with acute graft versus host disease (GvHD) 31 and autoimmune diseases such as systemic lupus erythematosus. Currently, it has not been established whether putative antiangiogenic MSCs can exert immunomodulatory properties or indeed influence hematopoiesis. But given that neovascularization plays a significant role in GvHD severity, for example, there is potential that treatment with antiangiogenic MSC populations could exert dual effects to both control pathological vascularization and provide immunomodulation.

The ongoing discrepancies described between stem cell studies could be due to MSC behavior in vitro not accurately representing their behavior in vivo. For example, BM‐MSCs have been shown in vivo to actually possess limited multipotency within bone, with genetic pulse chase studies of osteolineage progenitors demonstrating that in situ, BM‐MSCs have a hierarchical organization serving to replace short‐lived, postmitotic mature cells 32. These studies highlight that multipotency ex vivo should be interpreted with caution and are unlikely to represent how cells will function in vivo. Such observations should be extended to in vitro studies of MSC interactions with ECs, not representing how they interacted in vivo nor how they will behave if cultured and transplanted. It is also likely that extraction techniques and passage number used will be critical in disclosing any tissue‐specific differences present between MSC populations and their communication with the vasculature. Details of passage numbers and isolation techniques are frequently omitted from manuscript methodology, and often MSC populations are expanded continuously and become homogeneous. The species from which MSC populations have originated may have some influence on the angiogenic potential of the cells, with human MSCs appearing to exert the most antiangiogenic influence both in vitro and in vivo in contrast to mouse‐derived MCS, which appear to promote consistently proangiogenic effects (Table 1). This may also be linked to the age of the MSCs in these studies, with human MSCs generally being extracted from adult/aged tissue. Thus, direct comparison between studies and between tissues remains problematic. However, given that there has already been partial success in using MSCs in vivo to modulate angiogenesis, an improved understanding of MSC heterogeneity could further develop and broaden their use clinically. With aberrant vascular function central to many diseases, a capacity to control pathological angiogenesis using MSCs administered at certain densities, derived from specific tissues or cultured under specific spatial conditions, could provide exciting new opportunities to expand the use of MSCs therapeutically.

**Table 1:** Summary of angiogenic versus antiangiogenic influence of mesenchymal stem cells extracted from different species and tissues

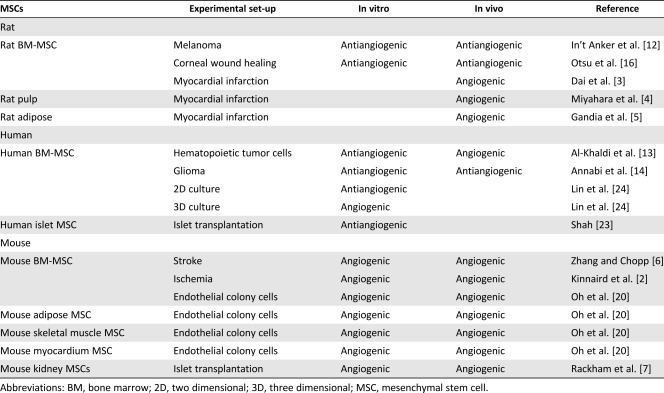

## Author Contributions

S.J.M.‐S. and C.E.C.: manuscript writing, final approval of the manuscript.

## Disclosure of Potential Conflicts of Interest

The authors indicated no potential conflicts of interest.
